# Supplementary material for: The potential of epigenetic therapy to target the 3D epigenome in endocrine-resistant breast cancer
Source: Nat Struct Mol Biol. 2024 Jan 5;31(3):498–512. doi: 10.1038/s41594-023-01181-7 (PMC10948365; doi:10.1038/s41594-023-01181-7)
Supplement: Supplementary file 1 — Supplementary Notes, Supplementary Methods, Supplementary References, Supplementary Figs. 1–6. [file 41594_2023_1181_MOESM1_ESM.pdf]

nature structural & molecular biology

Article https://doi.org/10.1038/s41594-023-01181-7

The potential of epigenetic therapy to target the 3D epigenome in endocrine-resistant breast cancer

In the format provided by the authors and unedited

The potential of epigenetic therapy to target the 3D epigenome in endocrine-resistant breast cancer

Supplementary Notes

Table of Contents

High degree of intra-tumour clonal heterogeneity is retained following Decitabine treatment 2

Decitabine-induced DNA hypomethylation activates transposable element (TE) expression....... 4

Multi-omics analyses of ER+ breast cancer PDX tumours following Decitabine treatment....... 5

Unsupervised clustering of multi-omics data 5

Quality control of Hi-C data 6

Relationship between differential TADs and A/B compartment switching 6

Relationship between differential TADs, differential interactions, DNA methylation and gene expression....... 6

Functional studies of DNA hypomethylation and re-methylation in TAMR cells.8

Supplementary Methods 8

Whole Genome Bisulphite Sequencing 8

WGBS analyses 8

Subclonal reconstruction from WGBS data....... 9

Somatic Copy Number Variation (sCNV) analyses 9

Cellular viability assay....... 10

Protein extraction and immunoblot analyses 10

Supplementary References....... 11

Supplementary Figures 14

Supplementary Figure 1 15

Supplementary Figure 2....... 19

Supplementary Figure 3....... 21

Supplementary Figure 4....... 23

Supplementary Figure 5....... 24

Supplementary Figure 6....... 25

High degree of intra-tumour clonal heterogeneity is retained following Decitabine treatment

Long-term anti-cancer treatments can act as selective forces that drive clonal evolution in the tumor, characterized by the outgrowth of rare cell populations that develop resistance. These surviving colonies have molecularly and functionally diverse resistant phenotypes, which are predetermined by intrinsic differences between the cells before drug exposure[1,2]. To first establish if the PDX tumours, following low-dose Decitabine treatment, retain the intra-tumour clonal heterogeneity or affected by clonal expansion, we tracked clonal dynamics after Decitabine treatment in the Gar15-13 and HCI-005 tumours using genetic and epigenetic approaches.

Somatic copy number variations (sCNVs) have been previously used for clonal reconstruction based on the overall CNV concordance between tumour samples[3,4]. First, we first inferred genome-wide sCNVs from the DNA methylation datasets (EPIC and WGBS) and Hi-C (see Supplementary Methods) in Vehicle- and Decitabine-treated PDX samples (Gar15-13 and HCI-005) used in the main study of the paper. CNV calls were generated first from EPIC DNA methylation data using conumee R package (v.19.0)[5] based on the signal intensity ratio between tumor and normal samples. To ensure that conumme can robustly capture accurate CNV information, we confirmed presence of all identified CNVs in WGBS (HCI-005 model) and Hi-C data with HiNT[6] (Gar15-13 model) (Supplementary Table 1). We then assessed clonality changes between Vehicle- and Decitabine-treated samples based on overall CNV concordance between samples, incorporating breakpoint locations. We detected 35 CNVs in Gar15-13 PDX tumours and 72 CNVs in HCI-005 PDX tumours that were commonly present in both Vehicle and Decitabine-treated tumours (Supplementary Table 1, Jaccard index: 0.979 and 0.937 for Gar15-13 and HCI-005, respectively), including a key amplification in Gar15-13 PDX model on chromosome 11 that encompasses the *CCND1* gene (Supplementary Fig. 1a) and large amplification on chromosome 8 that encompasses the *MYC* gene in HCI-005 model (Supplementary Fig. 1b). Notably, genome-wide we did not observe any significant changes in CNVs in the Decitabine-treated tumours, indicating no obvious clonal selection (Supplementary Fig. 1c-d).

Second, we performed a single-nucleotide variants (SNV) analyses from whole genome bisulphite sequencing (WGBS) data to assess the impact of Decitabine treatment on the clonal diversity. WGBS data was used to identify SNVs (see Supplementary Methods) and quantify their allele frequencies between Vehicle- and Decitabine-treated tumours.

We calculated variant allele frequencies (VAF) for identified SNVs (Supplementary Table 1) and compared VAF distribution between Vehicle- and Decitabine-treated samples. The distribution of VAFs of acquired SNVs in each tumour sample informs on the clonality of the cell population[7,8]. Monoclonal SNVs are those with a VAF distribution centred at ~0.5, while polyclonal SNVs, are those present in a small subpopulation, are characterized by a VAF of <0.25[7,8]. Overall, we found relatively stable clonal diversity across samples (Supplementary Fig. 1e-f), although some SNVs decreased or increased in VAF between Vehicle- and Decitabine-treated tumours, implying some degree of clonal evolution (Supplementary Fig. 1g-h). For instance, in Gar15-13 PDX model, SNVs in Vehicle-treated tumours are derived from a polyclonal population, translating into main VAF peak distributed around 0.3, while Decitabine-treated tumours contained a higher fraction of polyclonal SNVs, with main VAF peak distributed around 0.16 (Supplementary Fig. 1g), suggesting increase in the clonal heterogeneity.

Finally, recent studies used epigenomic data to study clonal dynamics[9], demonstrating high correlation between intra-tumour DNA methylation heterogeneity and genetic clonal heterogeneity[10,11]. These studies utilize computational methods that extract DNA methylation states co-occurring in a single sequencing read and consider each pattern as a pseudo-barcode that identifies each cell. By measuring the diversity of these patterns aligned at each genomic region, we can obtain a partial estimate of the true epigenetic diversity across cell population[12]. To perform clonal reconstruction based on DNA methylation heterogeneity we used methclone[13] and Metheor[14] (see Supplementary Methods). First, we compared epiallele composition between Decitabine- and Vehicle-treated samples (Gar15-13 and HCI-005) with methclone (min. read = 60, n. CpGs = 4). We used colours to define the 16 patterns of epialleles at four loci, where `0' stands for an un-methylated CpG and `1' stands for a methylated CpG. We found that the epiallele spectrum was maintained after Decitabine treatment, with an average of ~95 % (S.D. = 4.32 - 5.44%) of epialleles detected in Vehicle tumours sustained after treatment, defined as having combinatorial entropy from 0 to -20 (as per [13]) (Supplementary Fig. 1i-j). We then quantified epigenetic heterogeneity by calculating PDR and Epipolymorphism scores in Vehicle and Decitabine samples. We found that Decitabine and Vehicle samples have high levels of DNA methylation

heterogeneity, with significantly higher degree of DNA methylation disorder observed in response to Decitabine observed in both PDX models (Supplementary Fig. 1k-l). Together, these results demonstrate that in both Gar15-13 and HCI-005 PDX models, a high degree of intra-tumour clonal heterogeneity is retained following Decitabine treatment, with only a minor change to the clonal composition of the tumour's cell population observed.

Decitabine-induced DNA hypomethylation activates transposable element (TE) expression

Epigenetic therapies that target repressive epigenetic machinery have also been reported to mediate their anti-tumour effect by activating transposable elements (TEs) *via* the viral mimicry response[15,16]. Therefore, to study the viral mimicry response to Decitabine in our models, we first evaluated genome-wide methylation levels at different classes of repetitive elements using the REMP package[17], including LTRs, LINE1 elements and Alu elements. We observed genome-wide loss of DNA methylation at all repeat element sub-groups (Supplementary Fig. 2a, b). All repeat element classes showed ~12% loss of DNA methylation in Decitabine treated tumours. Notably, although all TE classes showed some degree of DNA methylation loss, the extent of DNA hypomethylation measured at repeat elements was less than those observed genome-wide and significantly less to DNA hypomethylation at enhancer regions (Extended Data Fig. 1h).

To assess whether Decitabine-induced hypomethylation increased transcription of TEs, we quantified global TE expression in poly-A enriched RNA-seq data using TEtranscripts[18] in four replicates of Vehicle and Decitabine-treated tumours. We detected a total of ~1079 TE transcripts in our samples (1049 in Gar15-13 and 1110 in HCI-005). Differential analysis of TE expression (DESeq2) revealed an increase in TE expression in both PDX models following treatment with Decitabine (Supplementary Fig. 2c, d), in agreement with previous studies showing that DNA hypomethylation activates TE expression in other model systems[19]. In total, 116 and 68 TEs were induced in Decitabine-treated Gar15-13 and HCI-005 tumours, respectively ($logFC > 0.2$ and $FDR < 0.1$) (Supplementary Fig. 2c, d). In both PDX models, LTRs and LINE1 elements were the most abundant class of differentially expressed TEs, followed by DNA transposons (Supplementary Fig. 2e, f).

To assess how loss of DNA methylation affected related antiviral signalling pathways in the PDX tumours, we investigated gene expression changes beyond TEs in Gar15-13 PDX tumours. Gene set enrichment analysis (GSEA) revealed that many immune pathways (inflammatory response, interferon response, IL6 signalling) were among the most upregulated in comparing Decitabine and Vehicle treated samples. Genes belonging to these pathways were up-regulated in Decitabine-treated tumours, including canonical components of the antiviral pathway (for example *CDKN1A*, *HIF1A* or *IFI27*) (Supplementary Fig. 2g, h), consistent with antiviral signalling induced by Decitabine.

Multi-omics analyses of ER+ breast cancer PDX tumours following Decitabine treatment

To provide a comprehensive characterisation of molecular changes following epigenetic therapy we used a multi-omics approach to generate and interrogate genome-wide maps of PDX breast tumours treated with and without Decitabine. We performed *in situ* Hi-C, promoter-anchored interactome (Promoter Capture Hi-C (PCHi-C)), DNA methylation (EPIC Microarrays), ER binding (ChIP-seq), H3K27ac binding (Cut&Run), CTCF binding (Cut&Run) and gene expression profiles (RNA-seq) in the same tumours. Together, we generated 69 libraries and more than 9.5 billion sequencing reads for Hi-C (Supplementary Table 5), PCHi-C (Supplementary Table 9), RNA-seq (Supplementary Table 6), ChIP-seq (Supplementary Table 4) and CUT&RUN (Supplementary Table 4).

Unsupervised clustering of multi-omics data

We initially explored the inter-subpopulation variability across the multi-omics datasets in the two PDX models (Gar15-13 and HCI-005) to determine if Decitabine treatment resulted in changes to high-level genome architecture. By comparing the top 10,000 most variable interactions between the Hi-C maps, we observed clear difference between Vehicle and Decitabine-treated tumours (Supplementary Fig. 3a), suggesting large-scale changes in the overall genome architecture. Furthermore, we interrogated Hi-C data from two endocrine-sensitive MCF7 cell lines and MCF7 tamoxifen-resistant derivative (TAMR) cell lines from [20] as well as the MCF7 cell line from [21] and performed MDS analysis. The comparison of samples shows that Decitabine treatment

induced a 3D genome organisation that is more similar to endocrine-sensitive MCF7 cells and clearly different from Vehicle-treated tumours, which clustered more closely to the tamoxifen-resistant TAMR cell line (Supplementary Fig. 3a). Principal component analyses (PCA) of the promoter-anchored chromatin interactions identified by ChICAGO from Promoter Capture Hi-C (PCHi-C) data (Supplementary Fig. 3b), DNA methylation (Supplementary Fig. 3c), ER binding (ChIP-seq) (Supplementary Fig. 3d), gene expression profiles (Supplementary Fig. 3e), H3K27ac binding (Cut&Run) (Supplementary Fig. 3f) and CTCF binding (Cut&Run) (Supplementary Fig. 3g) further confirmed large-scale differences were also induced in the methylome, TF binding, enhancer histone modifications and expression by Decitabine treatment.

Quality control of Hi-C data

The generated Hi-C data was of high quality in the low-input, snap-frozen PDX tumour tissues, as characterised by the high percentage of valid interactions (~90%), high cis/trans ratio (~80:20%) and high percentage of long-range interactions (60% >10KBs) (Supplementary Table 5 and Supplementary Fig. 4a). Host mouse reads were quantified (Supplementary Fig. 4b) and removed as described in [22]. We also observed strong concordance between Hi-C replicates as measured by the HiCRep SCC score [23] (Supplementary Fig. 4c).

Relationship between differential TADs and A/B compartment switching

The majority of differential TAD boundaries were located within stable A-type or B-type compartments (>95%) (Supplementary Fig. 4d), while a small number of lost TAD boundaries were located at regions that shifted from B-type to A-type assignment (81 TAD boundaries; Supplementary Fig. 4d).

Relationship between differential TADs, differential interactions, DNA methylation and gene expression

Although TADs were first proposed to serve as regulatory units for controlling gene expression by promoting and constraining long-range enhancer–promoter interactions (e.g. Schoenfelder and Fraser 2019[24]), recent work re-examined the relationship between gene regulation and TADs by observing that disruption of TAD features can alter expression for only a small number of genes (Despang et al. 2019[25]; Ghavi-Helm et al. 2019[26], reviewed in [27]). We analysed the association between differential TADs ("Overlapping TADs", "Decitabine-specific TADs" and "Vehicle-specific TADs") and

(1) differential interactions (Supplementary Fig. 4e), differentially methylated regions (DMRs) (Supplementary Fig. 4f) and differentially expressed genes (DEG) (Supplementary Fig. 4f) in our data by performing observed/expected analyses using permutation test (n = 1000). We found that differential interactions were significantly enriched at Decitabine-specific TAD boundaries, but none of the other observed associations were statistically significant (*P* value > 0.01).

Detection of differential promoter-anchored interactions from PCHi-C data

Promoter Capture Hi-C (PCHi-C) was performed as described previously [24,28,29]. Hi-C libraries were hybridised to custom-designed genomic restriction fragments covering 23,711 annotated gene promoters. We generated ~740,000,000 total valid read-pairs (di-tags) from the three PDX tumours for each treatment group, obtaining ~125 million valid ligation products per sample (~350 – 400 million per treatment) (Supplementary Table 9). To identify statistically significant interactions between promoters and other regulatory elements from the PCHi-C data, we used the CHiCAGO pipeline [30]. We obtained on average >100,000 statistically significant interactions per replicate. Identified interactions were highly enriched for histone modifications associated with open chromatin and regulatory elements (publicly available ChIP-seq datasets: H3K4me3, H3K4me1 and H3K27ac) as compared to random regions (Supplementary Fig. 4a, b). Concordance between individual Gar15-13 PDX tumour replicates of the PCHi-C data was confirmed by comparing the genomic locations of ChICAGO-called interactions in each PDX tumour replicate (min. score = 5, bin = 1.5Kb), demonstrating high overlap between significant interactions identified in each sample (Supplementary Fig. 4c).

To identify differential promoter-anchored interactions, we first used Chicdiff to calculate the asinh-transformed CHiCAGO scores and log fold changes between Vehicle and Decitabine tumours for each CHiCAGO interaction. We next integrated the Chicdiff scores with an intersection of CHiCAGO interactions identified in Vehicle and in Decitabine tumours and defined gained, maintained and lost interacting regions separated into promoter baits and enhancer OEs.

Functional studies of DNA hypomethylation and re-methylation in TAMR cells

First, to determine appropriate Decitabine dose, cytotoxicity was assessed using alamarBlue assay and 100nM dose was selected for subsequent experiments. Tamoxifen-resistant (TAMR) MCF7 cells were seeded in a white flat-bottom 96-well plate at a density of 2500 cells/well based on doubling time and allowed to adhere overnight. Cells were treated daily with Decitabine (0nM, 10nM, 50nM, 100nM, 200nM, 300nM, 500nM, 1uM, 5uM, 10uM and 100uM) for 72h and cellular viability was assessed on day 5, following 2 days of recovery using the alamarBlue (ThermoFisher, DAL1025) assay in accordance with the manufacture's recommendations. The normalised fluorescence intensity (A570/EM590) was calculated by subtracting the average background fluorescence intensity detected in wells containing media + 10% alamarBlue and no cells from the fluorescence intensity detected in wells containing cells. Based on cellular viability assayed, 100nM Decitabine dose was selected for further experiments (Supplementary Fig. 5a). Cells were then treated daily with decitabine (100 nM) for 7 consecutive days (Day 7 Decitabine samples). For Decitabine Recovery samples, after 7 days of Decitabine treatment, fresh media was added, and cells were cultured for 28 additional days and harvested on day 35. Control cells were collected at 11 days (Control Early) and 35 days (Control Late). Protein, DNA and RNA were harvested for Day 7 Decitabine, Decitabine Recovery and Control Early and Late samples. Loss and recovery of DNMT1 protein expression was confirmed by Western blot (Supplementary Fig. 5b).

Supplementary Methods

Whole Genome Bisulphite Sequencing

DNA from two Decitabine and two Vehicle-treated tumours from Gar15-13 PDX model was isolated from snap-frozen tumour samples using the Qiagen QIAamp DNA Mini Kit. WGBS experiments were performed using 150ng of bisulphite-converted DNA (EZ DNA Methylation Lighting kit (D5030, Zymo Research) using Accel-NGS Methyl-Seq DNA Library kit (DL-ILMMS-12) as described previously [31].

WGBS analyses

Raw WGBS reads were first trimmed to remove adaptor sequences using Trim Galore (v. 0.2.8) in paired-end mode and aligned to the human genome (hg38) using Bismark[32] (v. 0.24.0). The generated bam files were marked duplication by Picard tools (v.2.3.0)

and manipulated with samtools (v.1.2). Epipolymorphisms were computed from WGBS using methclone[13]. Four consecutive CpG sites (CpG quartets) that are supported by more than twenty sequencing reads were only considered. CpG quartets harboring CpG site that overlaps with dbSNP build 155 SNPs were excluded. For each CpG quartets, epipolymorphism was defined considering 16 possible patterns of DNA methylation states, with unmethylated and methylated states denoted as '0' and '1', respectively. Proportion of discordant reads (PDR) was computed using Metheor v0.1.2[14] to assess the fraction of reads carrying CpGs in discordant methylation states (i.e. containing both methylated and unmethylated CpGs in a single read) with respect to all reads mapped to a CpG.

Subclonal reconstruction from WGBS data

Subclonal reconstruction was performed using the approaches described in [7] from DNA sequencing data (WGBS) from two Decitabine- and two Vehicle-treated Gar15-13 and two Vehicle-treated and Decitabine-treated HCI-005 PDX tumours. Raw data from two biological replicates was merged to improve sensitivity and specificity of variant detection and to increase the limit of detection for minor subclones. First, single nucleotide variants (SNVs) were called using BISCUIT 1.2.0 (https://huishenlab.github.io/biscuit/), excluding SNVs in the CpG context and those on chromosome Y. SNVs present in dbSNP build 152 were considered as germline and the remaining SNVs were considered as somatic. Modified version of sCNAphase[33] pipeline was used for CNA reconstruction and to estimate tumor purity for all samples at germline variants from 22 autosomes. The pipeline incorporated a new function to simulate a normal sequencing sample in order to pair it with Decitabine and Vehicle samples to identify copy number alterations. We calculated clonal variant allele frequencies (VAF) for each variant by assessing the ratio of reference to alternative allele reads. Finally, we inspected the sub-clonal division based on the VAF distribution of the A/T->T/A somatic variants within the copy number neutral regions. VAF density was plotted with kernel density estimation and peak VAF point was estimated from local maxima of the difference using run-length encoding.

Somatic Copy Number Variation (sCNV) analyses

"Conumee: Enhanced copy-number variation analysis using Illumina DNA methylation arrays" R package version 1.9.0 was used to perform copy-number variation (CNV) analysis using Illumina EPIC methylation arrays. To identify copy

number variations from Hi-C data, we used HiNT (Hi-C for copy Number variation and Translocation detection)[6] method, which detects copy number variations within Hi-C data with breakpoints at single base-pair resolution. HiNT-CNV was run directly on Hi-C matrixes converted into .cool format using hicConvertFormat function from HiCExplorer[34] using default parameters.

Transposable elements (TE) expression analyses

For analysis of TE expression, adapter-trimmed, human-only RNA-seq reads were mapped with STAR (v.2.7.7a), allowing for multimapping alignments (flags: --outFilterMultimapNmax 100 --outFilterMismatchNmax 100). Annotation GTF files for canonical genes were downloaded from Ensembl genome browser (v.102, GRCh38.p13 assembly) and TE annotation GTF file (GRCm38_Ensembl_rmsk_TE.gtf) was downloaded from TEtranscripts [18] website (http://hammelllab.labsites.cshl.edu/software/#TEtranscripts). Normalisation and differential expression was performed for all genes and TEs using DESeq2[35]. TEs with and FDR < 0.1 and logFC > 0.7 were considered differentially expressed in pair-wise comparisons. Volcano plots were generated using EnhancedVolcano R package (v.1.8.0)[36].

Cellular viability assay

Tamoxifen-resistant (TAMR) MCF7 cells were seeded in a white flat-bottom 96-well plate at a density of 2500 cells/well based on doubling time and allowed to adhere overnight. Cells were treated daily with Decitabine (0nM, 10nM, 50nM, 100nM, 200nM, 300nM, 500nM, 1uM, 5uM, 10uM and 100uM) for 72h and cellular viability was assessed on day 5, following 2 days of recovery using the alamarBlue (ThermoFisher, DAL1025) assay in accordance with the manufacture's recommendations.

Protein extraction and immunoblot analyses

Whole cell protein lysates were prepared by resuspending scaped cells in modified RIPA buffer (50 mM Tris-HCL pH 7.5, 150 mM NaCl, 1 mM EDTA, 1 mM NaF, 1% Igepal, 0.25% Sodium-deoxycholate) with protease inhibitors and incubated on ice for 60 min with vortexing every 10 min. Lysate was sonicated using the Bioruptor on the high setting, 6x cycles of 30sec on/ 30sec off and stored at −80°C. Protein concentration was determined using a BCA assay (Pierce, #23227) according to manufacturer's instructions. Protein lysate was prepared using NuPAGE® LDS sample buffer (Life

Technologies, NP0007) and NuPAGE® sample reducing agent (Life Technologies, NP0004) followed by heating at 95°C for 5 min. Samples were resolved by gel electrophoresis using the NuPage Bis-Tris 4%–12% precast gel system according to the manufacturer's instructions (Life Technologies). Western blot transfer was carried out according to the manufacturer's instructions with the SureLock X-cell system (ThermoFisher Scientific) using transfer buffer containing 20% methanol content. Antibody used was C-terminal DNMT1 – Abcam #ab92314; GAPDH – Invitrogen #AM4300. Western blots (WBs) were then treated with Western Lightning Plus-ECL (Perkin Elmer, #NEL104001EA) before developing on Super Rx Fuji Medical X-Ray Film (Fujifilm, #4741019289) using the Konica Tabletop X-Ray Film Processor (#SRX101A). Developed film was scanned using the Epson Perfection V800/850 scanner.

Supplementary References

1. Goyal, Y. *et al.* Diverse clonal fates emerge upon drug treatment of homogeneous cancer cells. *Nature* (2023).
2. Fennell, K.A. *et al.* Non-genetic determinants of malignant clonal fitness at single-cell resolution. *Nature* **601**, 125-131 (2022).
3. Waldman, F.M. *et al.* Chromosomal alterations in ductal carcinomas in situ and their in situ recurrences. *J Natl Cancer Inst* **92**, 313-20 (2000).
4. Biermann, J. *et al.* Clonal relatedness in tumour pairs of breast cancer patients. *Breast Cancer Res* **20**, 96 (2018).
5. Blecua, P. *et al.* Refinement of computational identification of somatic copy number alterations using DNA methylation microarrays illustrated in cancers of unknown primary. *Brief Bioinform* **23**(2022).
6. Wang, S. *et al.* HiNT: a computational method for detecting copy number variations and translocations from Hi-C data. *Genome Biol* **21**, 73 (2020).
7. Tarabichi, M. *et al.* A practical guide to cancer subclonal reconstruction from DNA sequencing. *Nat Methods* **18**, 144-155 (2021).
8. Coorens, T.H.H. *et al.* Extensive phylogenies of human development inferred from somatic mutations. *Nature* **597**, 387-392 (2021).
9. Guo, M., Peng, Y., Gao, A., Du, C. & Herman, J.G. Epigenetic heterogeneity in cancer. *Biomark Res* **7**, 23 (2019).
10. Brocks, D. *et al.* Intratumor DNA methylation heterogeneity reflects clonal evolution in aggressive prostate cancer. *Cell Rep* **8**, 798-806 (2014).
11. Pan, H. *et al.* Epigenomic evolution in diffuse large B-cell lymphomas. *Nat Commun* **6**, 6921 (2015).
12. Scherer, M. *et al.* Quantitative comparison of within-sample heterogeneity scores for DNA methylation data. *Nucleic Acids Res* **48**, e46 (2020).
13. Li, S. *et al.* Dynamic evolution of clonal epialleles revealed by methclone. *Genome Biol* **15**, 472 (2014).
14. Dohoon Lee, B.K., Jeewon Yang, Sun Kim. Metheor: Ultrafast DNA methylation heterogeneity calculation from bisulfite read alignments. *bioRxiv* (2022).

15. Roulois, D. *et al.* DNA-Demethylating Agents Target Colorectal Cancer Cells by Inducing Viral Mimicry by Endogenous Transcripts. *Cell* **162**, 961-973 (2015).
16. Mehdipour, P. *et al.* Epigenetic therapy induces transcription of inverted SINEs and ADAR1 dependency. *Nature* **588**, 169-+ (2020).
17. Zheng, Y. *et al.* Prediction of genome-wide DNA methylation in repetitive elements. *Nucleic Acids Res* **45**, 8697-8711 (2017).
18. Jin, Y., Tam, O.H., Paniagua, E. & Hammell, M. TEtranscripts: a package for including transposable elements in differential expression analysis of RNA-seq datasets. *Bioinformatics* **31**, 3593-3599 (2015).
19. de Cubas, A.A. *et al.* DNA hypomethylation promotes transposable element expression and activation of immune signaling in renal cell cancer. *JCI Insight* **5**(2020).
20. Achinger-Kawecka, J. *et al.* Epigenetic reprogramming at estrogen-receptor binding sites alters 3D chromatin landscape in endocrine-resistant breast cancer. *Nat Commun* **11**, 320 (2020).
21. Barutcu, A.R. *et al.* Chromatin interaction analysis reveals changes in small chromosome and telomere clustering between epithelial and breast cancer cells. *Genome Biol* **16**, 214 (2015).
22. Dozmorov, M.G. *et al.* Chromatin conformation capture (Hi-C) sequencing of patient-derived xenografts: analysis guidelines. *Gigascience* **10**(2021).
23. Yang, T. *et al.* HiCRep: assessing the reproducibility of Hi-C data using a stratum-adjusted correlation coefficient. *Genome Research* **27**, 1939-1949 (2017).
24. Schoenfelder, S. & Fraser, P. Long-range enhancer-promoter contacts in gene expression control. *Nat Rev Genet* **20**, 437-455 (2019).
25. Despang, A. *et al.* Functional dissection of the Sox9-Kcnj2 locus identifies nonessential and instructive roles of TAD architecture. *Nat Genet* **51**, 1263-1271 (2019).
26. Ghavi-Helm, Y. *et al.* Highly rearranged chromosomes reveal uncoupling between genome topology and gene expression. *Nat Genet* **51**, 1272-1282 (2019).
27. de Wit, E. TADs as the Caller Calls Them. *J Mol Biol* **432**, 638-642 (2020).
28. Schoenfelder, S., Javierre, B.M., Furlan-Magaril, M., Wingett, S.W. & Fraser, P. Promoter Capture Hi-C: High-resolution, Genome-wide Profiling of Promoter Interactions. *Jove-Journal of Visualized Experiments* (2018).
29. Jung, I. *et al.* A compendium of promoter-centered long-range chromatin interactions in the human genome. *Nat Genet* **51**, 1442-1449 (2019).
30. Cairns, J. *et al.* CHiCAGO: robust detection of DNA looping interactions in Capture Hi-C data. *Genome Biol* **17**, 127 (2016).
31. Nair, S.S. *et al.* Guidelines for whole genome bisulphite sequencing of intact and FFPET DNA on the Illumina HiSeq X Ten. *Epigenetics & Chromatin* **11**(2018).
32. Krueger, F. & Andrews, S.R. Bismark: a flexible aligner and methylation caller for Bisulfite-Seq applications. *Bioinformatics* **27**, 1571-2 (2011).
33. Chen, W., Robertson, A.J., Ganesamoorthy, D. & Coin, L.J.M. sCNAphase: using haplotype resolved read depth to genotype somatic copy number alterations from low cellularity aneuploid tumors. *Nucleic Acids Res* **45**, e34 (2017).

34. Ramirez, F. *et al.* High-resolution TADs reveal DNA sequences underlying genome organization in flies. *Nat Commun* **9**, 189 (2018).
35. Love, M.I., Huber, W. & Anders, S. Moderated estimation of fold change and dispersion for RNA-seq data with DESeq2. *Genome Biology* **15**(2014).
36. Blighe K, S.R., M Lewis. EnhancedVolcano: Publication-ready volcano plots with enhanced colouring and labeling. (2018).

Supplementary Figures

Supplementary Figure 1

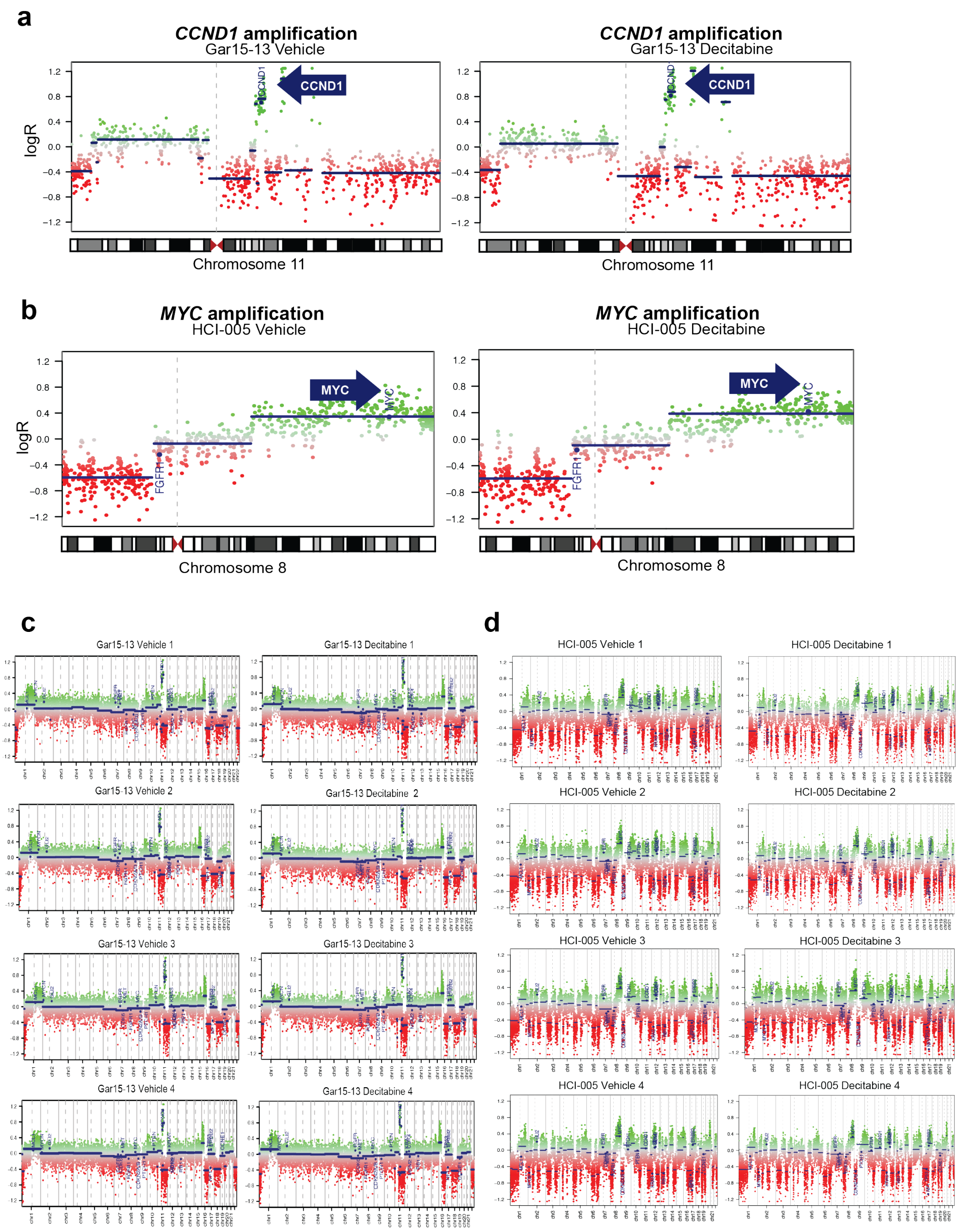

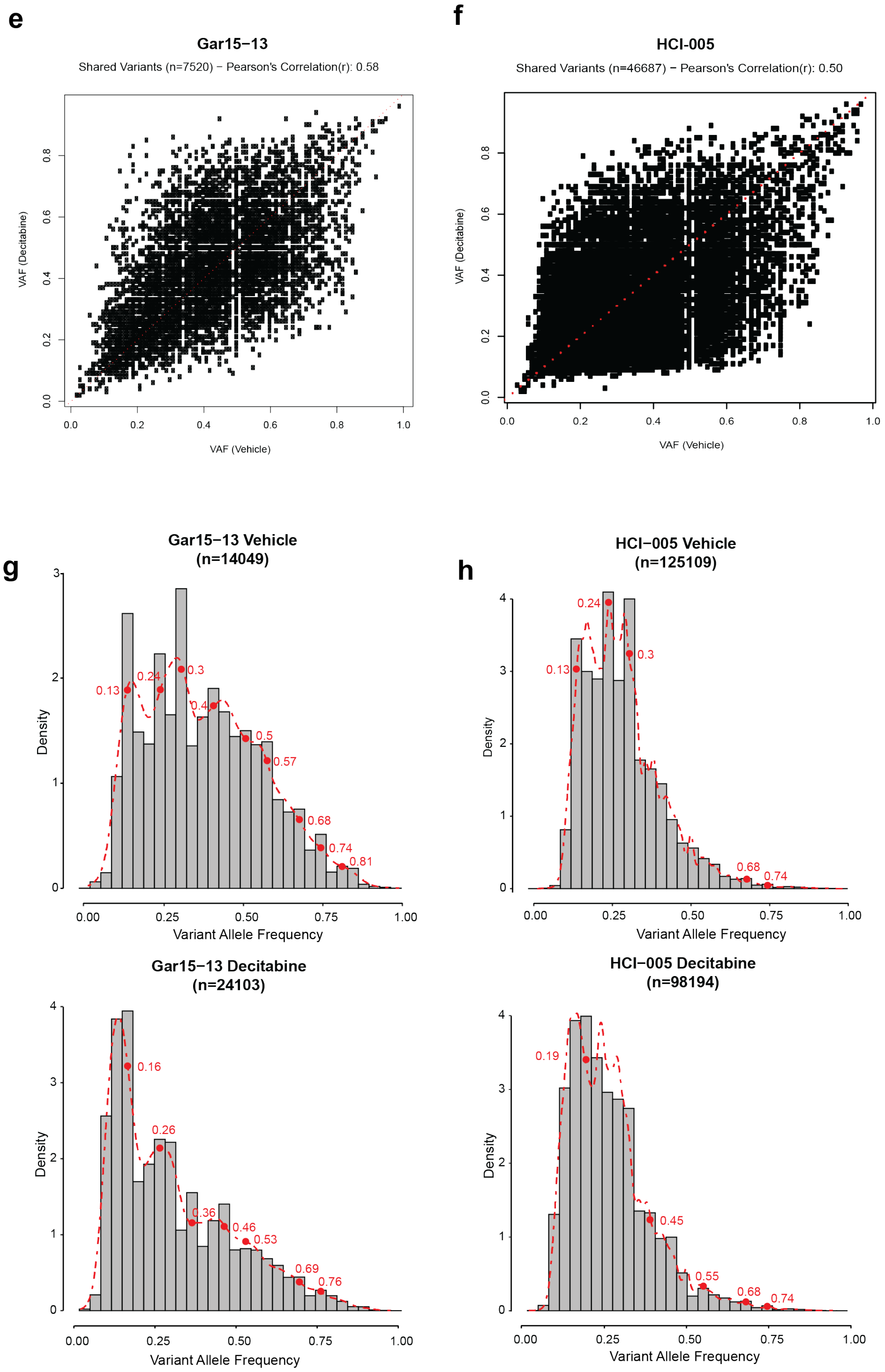
e
Gar15−13
Shared Variants (n=7520) − Pearson's Correlation(r): 0.58
VAF (Decitabine)
VAF (Vehicle)
f
HCI-005
Shared Variants (n=46687) − Pearson's Correlation(r): 0.50
VAF (Decitabine)
VAF (Vehicle)
g
Gar15−13 Vehicle
(n=14049)
Density
Variant Allele Frequency
h
HCI−005 Vehicle
(n=125109)
Density
Variant Allele Frequency
Gar15−13 Decitabine
(n=24103)
Density
Variant Allele Frequency
HCI−005 Decitabine
(n=98194)
Density
Variant Allele Frequency

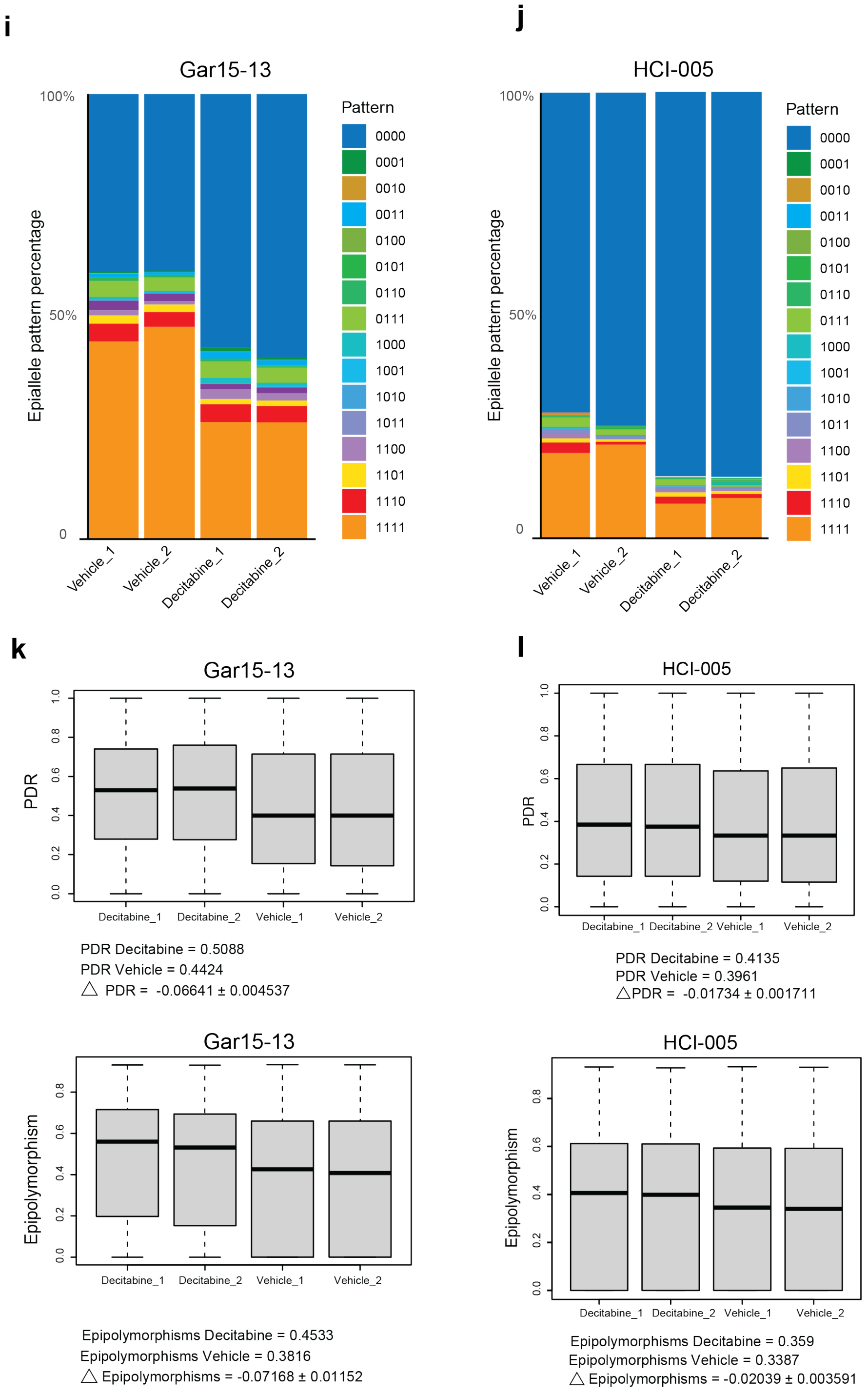
i
Gar15-13
100%
50%
0
Epiallele pattern percentage
Vehicle_1
Vehicle_2
Decitabine_1
Decitabine_2
Pattern
0000
0001
0010
0011
0100
0101
0110
0111
1000
1001
1010
1011
1100
1101
1110
1111
j
HCI-005
k
Gar15-13
PDR
Decitabine_1
Decitabine_2
Vehicle_1
Vehicle_2
PDR Decitabine = 0.5088
PDR Vehicle = 0.4424
△ PDR = -0.06641 ± 0.004537
l
HCI-005
PDR Decitabine = 0.4135
PDR Vehicle = 0.3961
△PDR = -0.01734 ± 0.001711
Gar15-13
Epipolymorphism
Epipolymorphisms Decitabine = 0.4533
Epipolymorphisms Vehicle = 0.3816
△ Epipolymorphisms = -0.07168 ± 0.01152
HCI-005
Epipolymorphism
Epipolymorphisms Decitabine = 0.359
Epipolymorphisms Vehicle = 0.3387
△ Epipolymorphisms = -0.02039 ± 0.003591

Supplementary Fig. 1

(a) Somatic CNV profile of chromosome 11 in Vehicle- and Decitabine-treated Gar15-13 PDX tumours. Zoomed-in view of the amplification encompassing the *CCND1* gene locus.
(b) Somatic CNV profile of chromosome 8 in Vehicle- and Decitabine-treated HCI-005 PDX tumours. Zoomed-in view of the amplification encompassing the *MYC* gene locus.
(c) Genome-wide somatic CNV profiles of Vehicle- and Decitabine-treated Gar15-13 PDX tumours (n = 4 biological replicates each).
(d) Genome-wide somatic CNV profiles of Vehicle- and Decitabine-treated HCI-005 PDX tumours (n = 4 biological replicates each).
(e) Density plots for the Gar15-13 PDX model. SNV VAFs from each Vehicle- and Decitabine treated tumours are plotted along the axes.
(f) Density plots for the HCI-005 PDX model. SNV VAFs from each Vehicle- and Decitabine treated tumours are plotted along the axes.
(g) VAF distribution histograms for Vehicle- and Decitabine-treated Gar15-13 PDX tumour samples. The number indicated in the title of each histogram is the SNV burden. The red dashed line indicates kernel density estimation with the estimated peak VAF indicated on the plot.
(h) VAF distribution histograms for Vehicle- and Decitabine-treated HCI-005 PDX tumour samples. The number indicated in the title of each histogram is the SNV burden. The red dashed line indicates kernel density estimation with the estimated peak VAF indicated on the plot.
(i) Epiallele pattern proportion in Vehicle- and Decitabine-treated Gar15-13 PDX tumours. Merged data from n = 2 biological replicates shown.
(j) Epiallele pattern proportion in Vehicle- and Decitabine-treated HCI-005 PDX tumours. Merged data from n = 2 biological replicates shown.
(k) Genome-wide PDR and Epipolymorphism comparison between Vehicle- and Decitabine treated Gar15-13 PDX tumours (n = 2 biological replicates each). The centre line denotes the median, the upper and lower box limits denote upper and lower quartiles, and the whiskers denote range.
(l) Genome-wide PDR and Epipolymorphism comparison between Vehicle- and Decitabine treated HCI-005 PDX tumours (n = 2 biological replicates each). The centre line denotes the median, the upper and lower box limits denote upper and lower quartiles, and the whiskers denote range.

Supplementary Figure 2

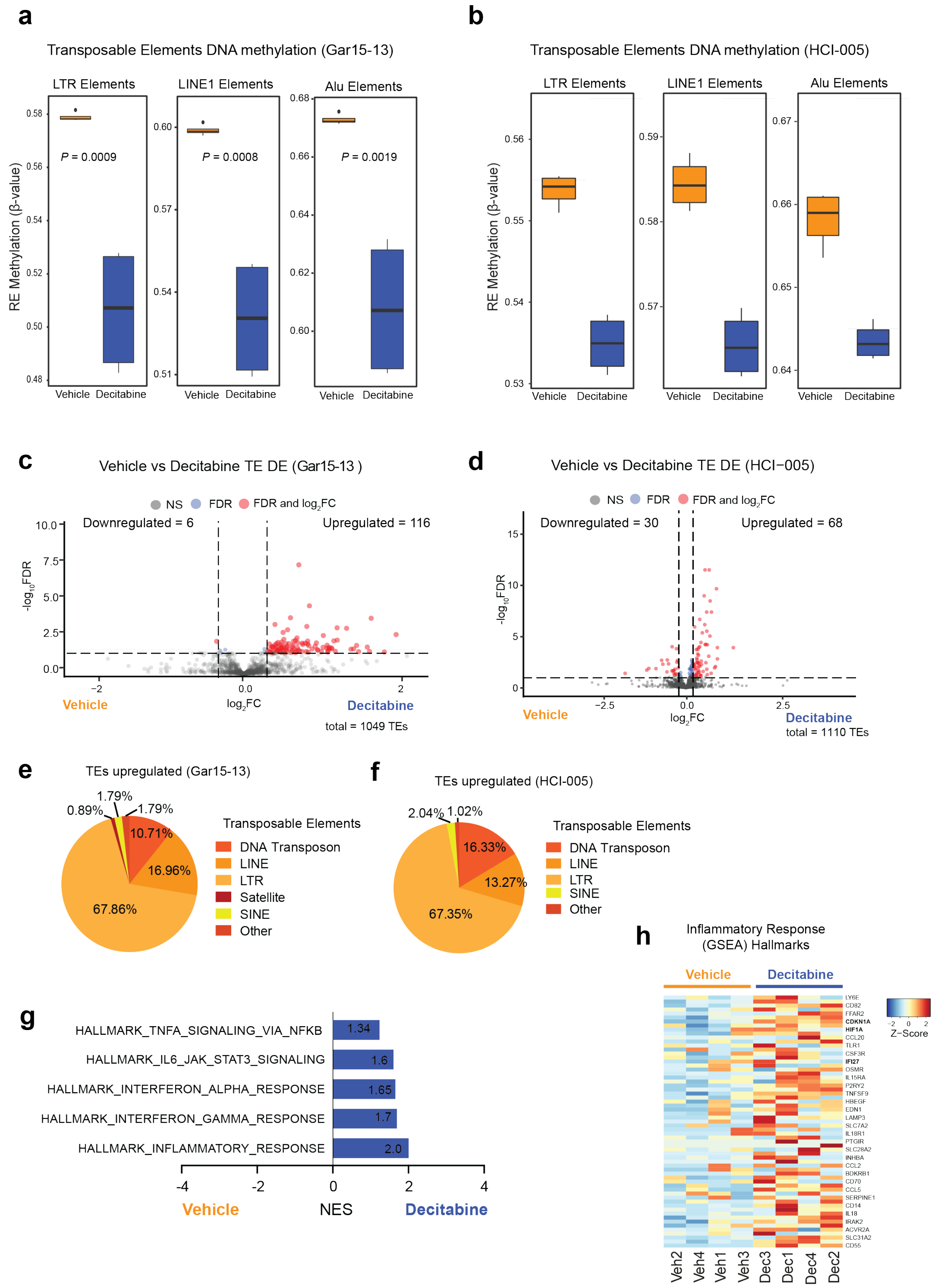

Supplementary Fig. 2

(a) Distribution of DNA methylation for Gar15-13 Vehicle and Decitabine-treated tumours for EPIC probes mapping to LTR, LINE1 and Alu repetitive elements (REMP annotation) (n = 2 technical replicates each). Black line indicates mean ± SD.

Box plots show median, inter-quartile range and maximum/minimum log fold change. Data analysed using the two-sided Z test.
(b) Distribution of DNA methylation for HCI-005 Vehicle and Decitabine-treated tumours for EPIC probes mapping to LTR, LINE1 and Alu repetitive elements (REMP annotation). n = 2 technical replicates. Black line indicates mean ± SD. Box plots show median, inter-quartile range and maximum/minimum log fold change. Data analysed using the two-sided Z test.
(c) Differential expression of transposable elements in Decitabine-treated Gar15-13 tumours compared to Vehicle. Lines indicate differentially expressed TEs considered significant (logFC > 0.2 and FDR < 0.1).
(d) Differential expression of transposable elements in Decitabine-treated HCI-005 tumours as compared to Vehicle. Lines indicate differentially expressed TEs considered significant (logFC > 0.2 and FDR < 0.1).
(e) Distribution of differentially expressed Transposable Elements (TE) classes in Gar15-13.
(f) Distribution of differentially expressed TE classes in HCI-005.
(g) Normalised enrichment scores (NES) for significantly enriched gene sets belonging to the immune GSEA hallmark in Gar15-13.
(h) RNA-seq heatmap of Decitabine-induced changes in expression of genes belonging to the Inflammatory Response (GSEA) Hallmarks in Gar15-13. Top differentially expressed genes plotted (FDR < 0.05).

Supplementary Figure 3

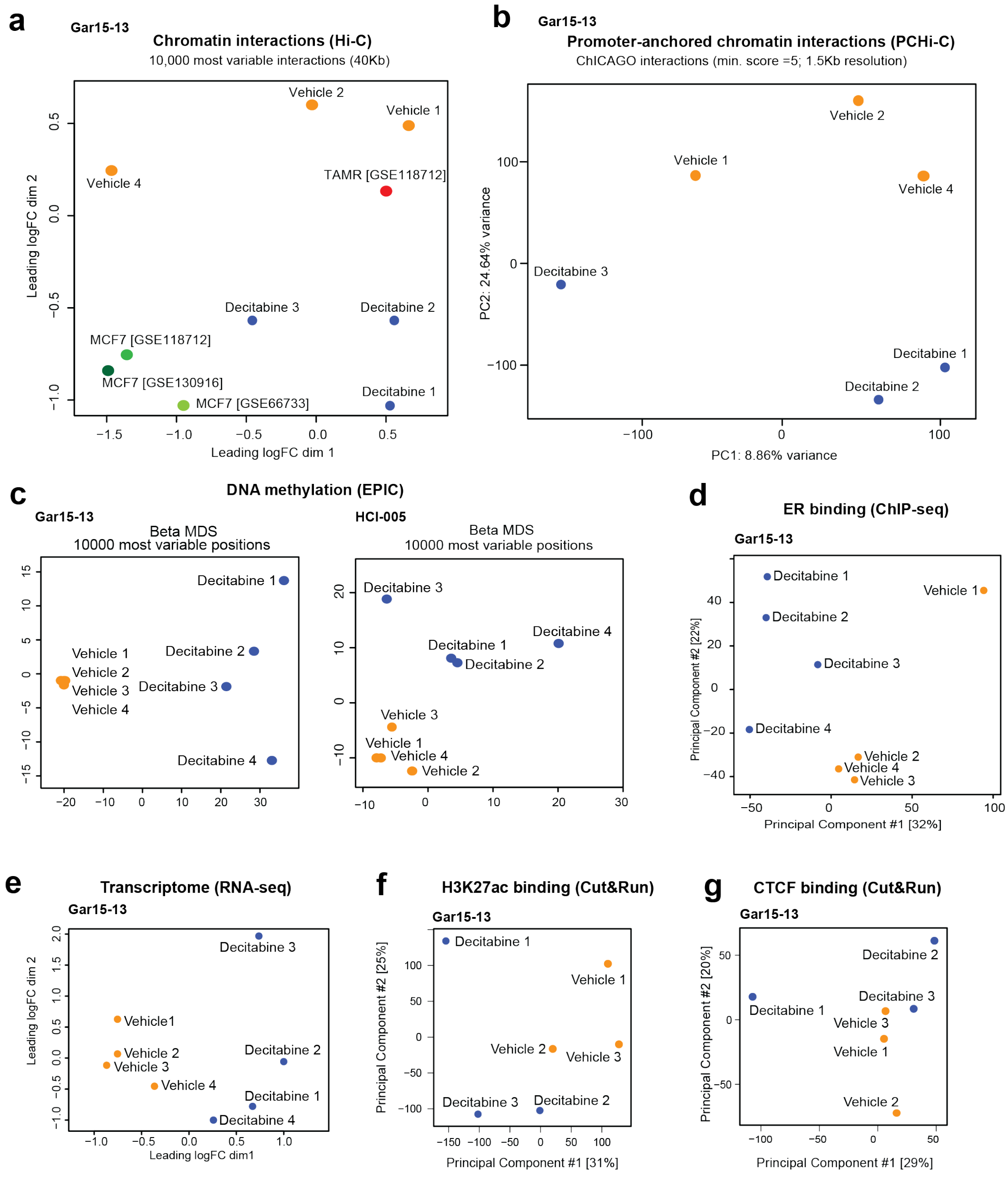

Supplementary Fig. 3

(a) Multidimensional-scaling plot showing the relationships among the chromatin interaction profiles of Gar15-13 Vehicle tumours, Decitabine-treated tumours (n = 3 biological replicates each), tamoxifen-resistant MCF7 cells (TAMR; n = 1 replicate) and parental MCF7 cells (MCF7; n = 2 replicates) and MCF7 cell lines from [21]. Distances on the plot represent the leading log fold change (logFC). Data plotted for top 1000 most variable interactions.

(b) Principal component analyses showing the relationship among the promoter-anchored chromatin interactions of Gar15-13 Vehicle and Decitabine-treated tumours (n = 3 biological replicates each). Data plotted for CHiCAGO-called interactions (min. score = 5) at 1.5Kb resolution.

(c) Principal component analysis showing the relationship among the DNA methylation profiles of Gar15-13 (left) and HCI-005 (right) Vehicle tumours, Decitabine-treated tumours (n = 4 biological replicates each). Data plotted for Beta values from top 10,000 most variable probes.
(d) Principal component analysis showing the relationship among the ER binding profiles of Gar15-13 Vehicle tumours and Decitabine-treated tumours (n = 4 biological replicates each). Data plotted for normalised read counts.
(e) Principal component analysis showing the relationship among the transcriptome profiles of Gar15-13 (left) Vehicle and Decitabine-treated tumours (n = 4 biological replicates each). Data plotted for normalised TPM values.
(f) Principal component analysis showing the relationship among the H3K27ac binding CUT&RUN profiles of Gar15-13 Vehicle and Decitabine-treated tumours (n = 3 biological replicates each). Data plotted for normalised read counts.
(g) Principal component analysis showing the relationship among the CTCF binding CUT&RUN profiles of Gar15-13 Vehicle and Decitabine-treated tumours (n = 3 biological replicates each). Data plotted for normalised read counts.

Supplementary Figure 4

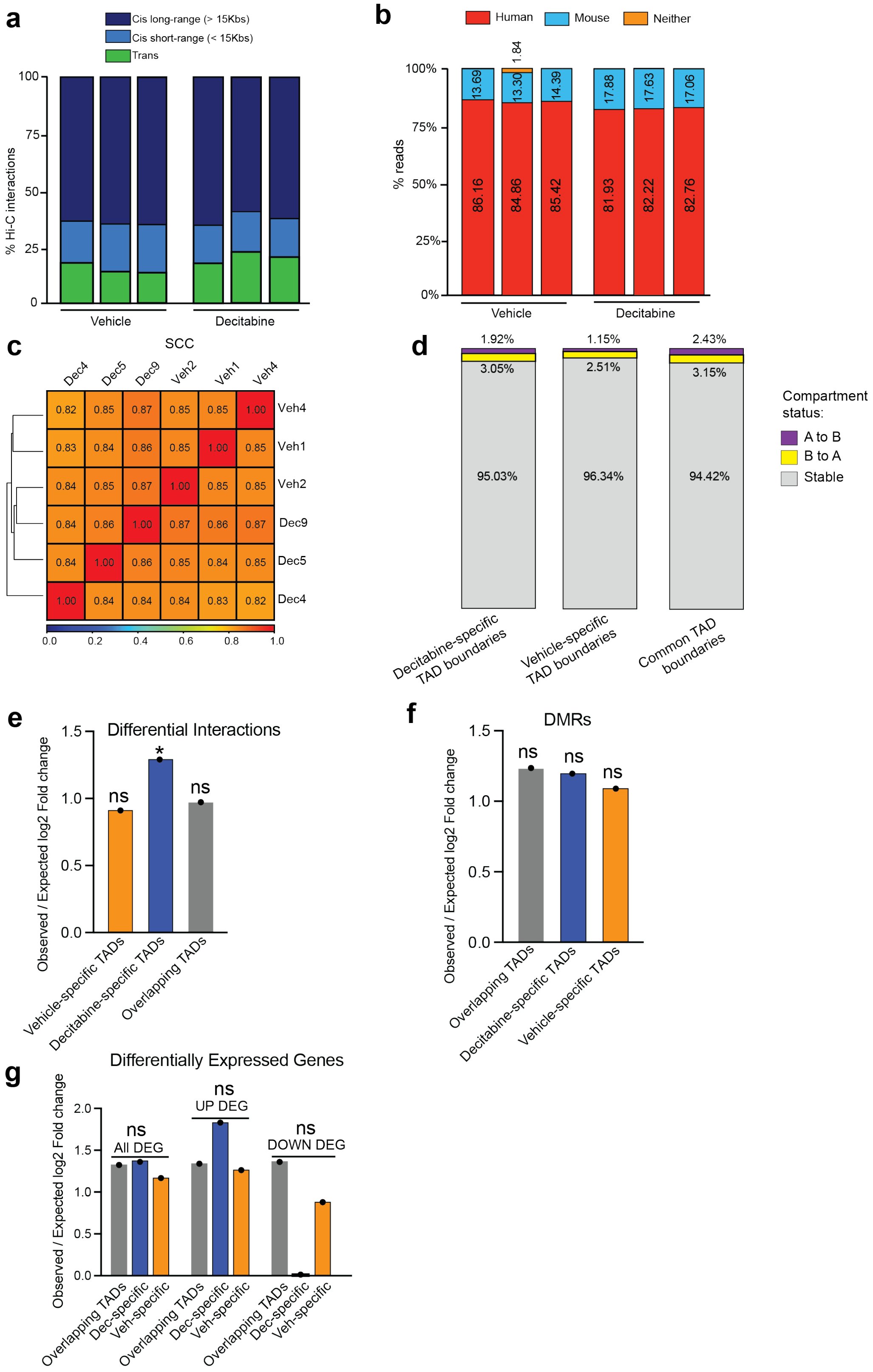

Supplementary Fig. 4

(a) Distribution of short- and long-range intra-chromosomal and inter-chromosomal interactions identified in *in situ* Hi-C data from Gar15-13 PDX tumours.
(b) Proportion of human (hg38) and mouse (mm10) reads detected by Xenome in Hi-C data from PDX tumours.
(c) Heatmap of stratum adjusted correlation coefficient (SCC) values calculated by HiCRep between the Hi-C interaction maps.
(d) Percentage overlap between differential (Decitabine-specific and Vehicle-specific) and common TAD boundaries and A/B compartment switching.
(e) Observed over expected fold change enrichment of differential interactions in Gar15-13 Decitabine treatment compared to Vehicle across overlapping and differential TAD boundaries. * P value < 0.001, ns – Not Significant (permutation test).
(f) Observed over expected fold change enrichment of differential DNA methylation (DMRs) in Gar15-13 Decitabine treatment compared to Vehicle across overlapping and differential TAD boundaries. ns – Not Significant, (permutation test).
(g) Observed over expected fold change enrichment of differentially expressed genes (DEG) in Gar15-13 Decitabine treatment compared to Vehicle across overlapping and differential TAD boundaries. ns – Not Significant (permutation test).

Supplementary Figure 5

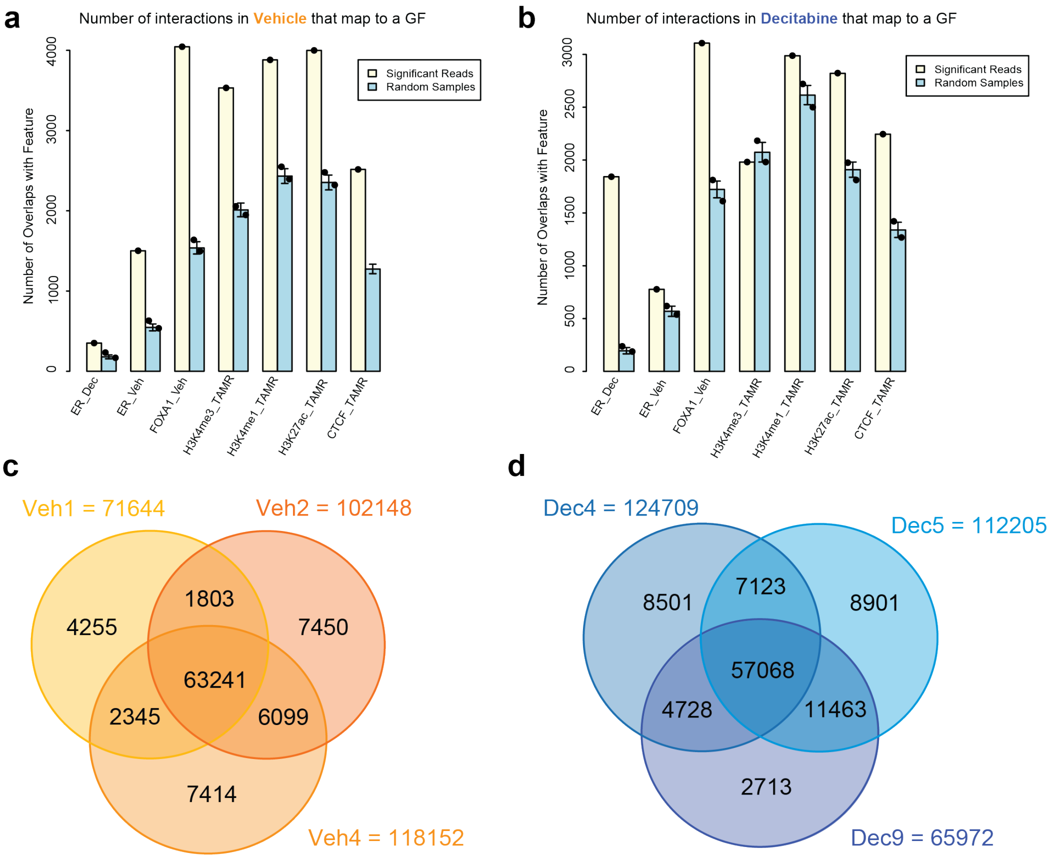

Supplementary Fig. 5

(a) Significant CHiCAGO promoter-anchored interactions in three Vehicle tumours enrichment for histone marks and transcription factors compared with distance-matched random regions. All data are represented as mean ± SD. Error bars show SD across 100 draws of random regions.
(b) Significant CHiCAGO promoter-anchored interactions in three Decitabine-treated tumours enrichment for histone marks and transcription factors compared with distance-matched random regions. All data are represented as mean ± SD. Error bars show SD across 100 draws of random regions.
(c) Overlap between CHiCAGO-called interactions (min. score = 5, bin = 1.5Kb) in Vehicle-treated Gar15-13 PDX tumour replicates of PCHi-C data.
(d) Overlap between CHiCAGO-called interactions (min. score = 5, bin = 1.5Kb) in Decitabine-treated Gar15-13 PDX tumour replicates of PCHi-C data.

Supplementary Figure 6

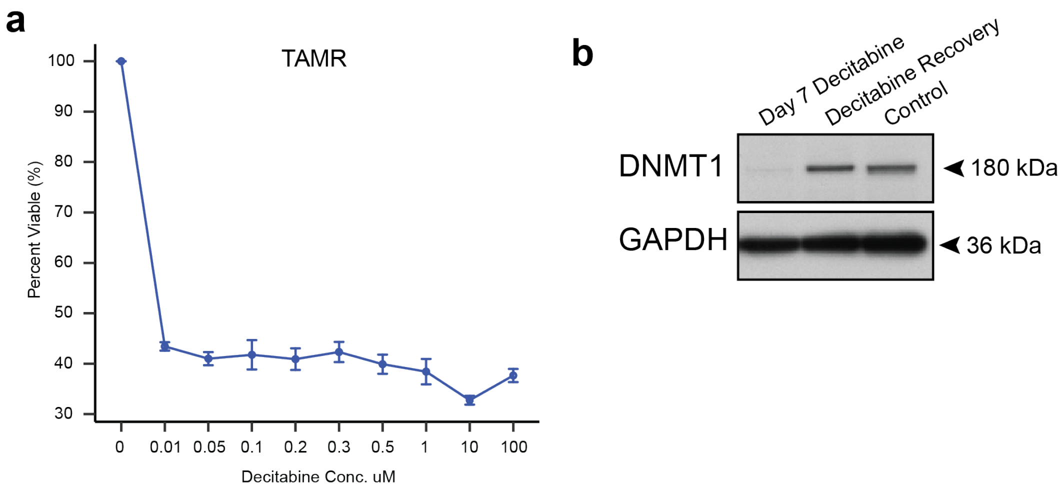

Supplementary Fig. 6

(a) Endocrine-resistant MCF7 cells (TAMRs) were treated with the indicated doses of decitabine for 3 consecutive days and assayed on day 5. Decitabine dose-response curve for viability in TAMR cells. All data are mean ± SD (n = 4 technical replicates).
(b) DNMT1 protein levels were assessed in TAMR cells at day 7 of Decitabine treatment (100nM) by immunoblot analysis. GAPDH was included as a loading control. Blots are representative of three experiments corresponding to independent passages of cells.
