# Supplementary material for: The potential of epigenetic therapy to target the 3D epigenome in endocrine-resistant breast cancer
Source: Nat Struct Mol Biol. 2024 Jan 5;31(3):498–512. doi: 10.1038/s41594-023-01181-7 (PMC10948365; doi:10.1038/s41594-023-01181-7)
Supplement: Supplementary file 2 — Reporting Summary [file 41594_2023_1181_MOESM2_ESM.pdf]

nature portfolio

Corresponding author(s): Susan J. Clark and Joanna Achinger-Kawecka

Last updated by author(s): 10/30/2023

Reporting Summary

Nature Portfolio wishes to improve the reproducibility of the work that we publish. This form provides structure for consistency and transparency in reporting. For further information on Nature Portfolio policies, see our Editorial Policies and the Editorial Policy Checklist.

Statistics

For all statistical analyses, confirm that the following items are present in the figure legend, table legend, main text, or Methods section.

n/a	Confirmed	
☐	☒	The exact sample size (*n*) for each experimental group/condition, given as a discrete number and unit of measurement
☐	☒	A statement on whether measurements were taken from distinct samples or whether the same sample was measured repeatedly
☐	☒	The statistical test(s) used AND whether they are one- or two-sided<br>*Only common tests should be described solely by name; describe more complex techniques in the Methods section.*
☒	☐	A description of all covariates tested
☐	☒	A description of any assumptions or corrections, such as tests of normality and adjustment for multiple comparisons
☐	☒	A full description of the statistical parameters including central tendency (e.g. means) or other basic estimates (e.g. regression coefficient) AND variation (e.g. standard deviation) or associated estimates of uncertainty (e.g. confidence intervals)
☐	☒	For null hypothesis testing, the test statistic (e.g. *F*, *t*, *r*) with confidence intervals, effect sizes, degrees of freedom and *P* value noted<br>*Give P values as exact values whenever suitable.*
☒	☐	For Bayesian analysis, information on the choice of priors and Markov chain Monte Carlo settings
☒	☐	For hierarchical and complex designs, identification of the appropriate level for tests and full reporting of outcomes
☐	☒	Estimates of effect sizes (e.g. Cohen's *d*, Pearson's *r*), indicating how they were calculated

Our web collection on statistics for biologists contains articles on many of the points above.

Software and code

Policy information about availability of computer code

Data collection

All data collection used open-access or commercially available software as outlined below:

The Illumina NextSeq 500, HiSeq X10 and NovaSeq S4 were used for RNA-seq, ChIP-seq, CUT&RUN, Hi-C and PCHi-C studies.
The Nanozoomer slide scanner was used to capture images of stained tissue sections.

Graphpad Prism 8 (Graphpad Software, Inc.)
minfi (v.1.34.0) (Aryee et al., 2014)
limma (v.3.46) (Ritchie et al., 2015)
conumee (v.1.9.0)
DMRcate (v.2.2.3) (Peters et al., 2015)
REMP (v.1.14.0) (Zheng et al., 2017)
FastQ Screen (v.0.14.1) (Wingett and Andrews, 2018)
Bismark (v.0.24.0) (Krueger et al., 2011)
methclone (v.0.1.0) (Li et al., 2014)
metheor (v.1) (Lee et al., 2022)
sCNAphase (Chen et al., 2017)
Xenome (v.1.0.1) (Conway et al., 2012)
HiC-Pro (v.2.11.4) (Servant et al., 2015)
Juicer (v.1.6) (Durand et al., 2016)
TADtool (v.0.76) (Kruse et al., 2016)
GENOVA (v.0.95) (van der Weide et al., 2021)
Homer (v.4.8) (Heinz et al., 2010)
HiCUP (v.0.7.4) (Wingett et al., 2015)
HiNT (v.2.2.7) (Wang et al., 2020)

CHiCAGO (v.1.14.0) (Cairns et al., 2016)
Chicdiff (v.0.6) (Cairns et al., 2019)
EnhancedVolcano (v.1.8.0) (Blighe et al., 2018)
STAR (v.2.7.7a) (Dobin et al., 2013)
edgeR (v.3.18.1) (Robinson et al., 2010)
bedtools (v.2.25) (Quinian and Hall, 2010)
TEtranscripts (v.2.2.1) (Jin et al., 2015)
Bowtie2 (v2.3.4.1) (Langmead and Salzberg, 2012)
TrimGalore (v0.6.10)
MACS2 (v2.2.6) (Zhang et al., 2008)
DESeq2 (v.1.3.0) (Love et al., 2014)
GAT (v.1.3.4) (Heger et al., 2013)
DiffBind (v.3.0.9) (Ross-Innes et al., 2012)
ChIPseeker (v.1.26.0) (Yu et al., 2015)
deepTools2 (v.3.5.0) (Ramirez et al., 2016)
GSEA (v.4.1.0)
MSigDB (v.7.2) (Subramanian et al., 2005)
cBioPortal (Cerami et al., 2012)
survminer (v.0.4.9)

Data analysis

All analyses were performed using open source software. All software code used to analyze the data for this study is publicly available as described in the methods section. Python script language (v.2.7.8 and v.3.9.1) and R (v.3.6.3 and v.4.0.3) were used for bioinformatics methods and algorithms in this work. All code for Hi-C, PCHi-C, ChIP-seq and RNA-seq analyses is publicly available within the GitHub repository https://github.com/JoannaAch/PDX_Decitabine_3DEpigenome.

For manuscripts utilizing custom algorithms or software that are central to the research but not yet described in published literature, software must be made available to editors and reviewers. We strongly encourage code deposition in a community repository (e.g. GitHub). See the Nature Portfolio guidelines for submitting code & software for further information.

Data

Policy information about availability of data

All manuscripts must include a data availability statement. This statement should provide the following information, where applicable:

- Accession codes, unique identifiers, or web links for publicly available datasets
- A description of any restrictions on data availability
- For clinical datasets or third party data, please ensure that the statement adheres to our policy

All sequencing data created in this study have been uploaded to the Gene Expression Omnibus (GEO; https://www.ncbi.nlm.nih.gov/geo/) and are available under primary accession code GSE171074 and GSE216989. Public datasets include: ChIP-seq data sets downloaded from GSE32222 by Ross-Innes et al., 2012, ChromHMM data downloaded from GSE118716 by Achinger-Kawecka et al., 2020. All data was mapped to hg38 human reference genome.

Field-specific reporting

Please select the one below that is the best fit for your research. If you are not sure, read the appropriate sections before making your selection.

☒ Life sciences ☐ Behavioural & social sciences ☐ Ecological, evolutionary & environmental sciences

For a reference copy of the document with all sections, see nature.com/documents/nr-reporting-summary-flat.pdf

Life sciences study design

All studies must disclose on these points even when the disclosure is negative.

Sample size

Patient-derived tumour xenograft (PDX) models generated from two different endocrine-resistant, metastatic ER+ breast cancer patients (Gar15-13 and HCI-005) were used to account for biological and clinical variability between patients. Decitabine treatment was performed on 8 (Gar15-13) and 7 (HCI-005) individual PDX mice to obtain sufficient sample size based on sample size calculation for standard statistical tests (80% statistical power to detect 1.3 SD difference and 95% power to detect 1.7 SD difference), with the exact number of replicates in the figure legends.

No statistical method was used to determine sample sizes in cell line experiments. Sample sizes were selected prior to knowledge of the outcome. No power analyses were carried out.

EPIC DNA methylation, ER ChIP-seq and RNA-seq experiments were performed in quadruplicates and Hi-C, Promoter Capture Hi-C and CUT&RUN were performed in triplicates to assess statistical significance.

EPIC DNA methylation, RNA-seq experiments and Promoter Capture Hi-C in TAMR cells were performed in duplicates.

Data exclusions

Sample sizes differed between in vivo xenograft tumour growth experiments, as some tumours did not grow at the expected rate. Such outliers were excluded from further data analyses.

Replication

Decitabine treatment was performed in two independent patient-derived xenografts (PDXs) and the tumour inhibiting effect of Decitabine

Replication	was replicated in both models, across multiple mice. Two unique tumour xenograft models (Gar15-13, HCI-005) were used in this study to ensure consistent responses across a variety of tumours. Most assays were performed in at least a biological triplicate. All experiments were able to be reliably reproduced.<br><br>Hi-C experiments were performed in triplicates and reproducibility between replicates was verified using HiCRep (Yang T (2018)). EPIC, RNA-seq and ER ChIP-seq experiments were performed in four replicates in 2 PDX models. All findings were reproducible and instances of variability are discussed in the text.<br><br>Final conclusions were validated in an independent cell line model of endocrine-resistance (TAMR) with 7 days of Decitabine treatment.
Randomization	PDX mice were randomised to treatment arms when tumours reached 200mm3 using an online randomisation tool (https://www.graphpad.com/quickcalcs/randomize1.cfm) (n = 6 - 8 mice per group for therapeutic studies, exact numbers specified in figure legends). Cells were randomly split from the same pool of cells before subject to treatments. Randomization was not applicable to other experiments.
Blinding	In vivo experiments utilized blinded animal technicians for assessing disease severity. The investigators were not blinded to the group allocation during data collection and outcome assessment. In order to analyse data and assign samples to the correct group, experimenters needed to be unblinded.

Reporting for specific materials, systems and methods

We require information from authors about some types of materials, experimental systems and methods used in many studies. Here, indicate whether each material, system or method listed is relevant to your study. If you are not sure if a list item applies to your research, read the appropriate section before selecting a response.

Materials & experimental systems

n/a	Involved in the study
☐	☒ Antibodies
☐	☒ Eukaryotic cell lines
☒	☐ Palaeontology and archaeology
☐	☒ Animals and other organisms
☒	☐ Human research participants
☒	☐ Clinical data
☒	☐ Dual use research of concern

Methods

n/a	Involved in the study
☐	☒ ChIP-seq
☒	☐ Flow cytometry
☒	☐ MRI-based neuroimaging

Antibodies

Antibodies used	ChIP-seq:<br>5ug Anti-Estrogen Receptor alpha (HC-20), Santa Cruz Biotechnology (Cat#sc-543; RRID: AB_631471)<br>CUT&RUN:<br>0.5ug CTCF (CTCF CUTANA™ CUT&RUN Antibody (cat. #13-2014))<br>0.5ug H3K27ac (Histone H3K27ac Antibody, SNAP-ChIP Certified (cat. #13-0045))<br>0.5ug IgG (CUTANA Rabbit IgG CUT&RUN Negative Control (cat. #13-0042))<br><br>IHC:<br>Monoclonal Mouse Anti-Human Estrogen Receptor α, Clone 1D5, Agilent (Cat# M7047, RRID:AB_2101946)<br>Monoclonal Mouse Anti-Human Ki-67 Antigen, Clone MIB-1, Agilent (Cat# M7240, RRID:AB_2142367)<br>Western blot:<br>C-terminal DNMT1 antibody, Abcam (Cat#ab92314) (1:1000)<br>GAPHD antibody, Invitrogen Antibodies (Cat#AM4300) (1:1000)
Validation	Validation of the antibodies was performed either through indirect validation through published literature (Hickey et al., Nat Medicine, 2021) or from the antibody manufacturers/distributors themselves.

Eukaryotic cell lines

Policy information about cell lines

Cell line source(s)	Parental MCF7 breast cancer cells and endocrine-resistant TAMR and FASR cells were obtained from our collaborator Dr Julia Gee (Cardiff University, UK).
Authentication	All cell lines were authenticated by short-tandem repeat profiling (CellBank Australia, Westmead, NSW, Australia) and cultured for <6 months after authentication.
Mycoplasma contamination	All cell lines used in-house tested negative for mycoplasma using the MycoAlert Mycoplasma Detection Kit (Lonza, #LT07-318).
Commonly misidentified lines<br>(See ICLAC register)	No cell lines from the ICLAC register were used.

Animals and other organisms

Policy information about studies involving animals; ARRIVE guidelines recommended for reporting animal research

Laboratory animals	6–8-week-old female NOD-scid IL2Rγnull (NSG) mice, obtained from Australian BioResources (Sydney, Australia) were used in the study. Mice were socially housed at the Garvan Institute of Medical Research specific pathogen free (SPF) animal facility, in temperature and light cycle-controlled rooms and given ad lib access to food, water and nesting materials.
Wild animals	The study did not involve wild animals.
Field-collected samples	The study did not involve samples collected from the field.
Ethics oversight	All in vivo experiments, procedures and endpoints were approved by the Garvan Institute of Medical Research Animal Ethics Committee (HREC #14/35, #15/25, ARA #21/11) and performed at the Garvan Institute of Medical Research using standard techniques in accordance with relevant national and international guidelines.

Note that full information on the approval of the study protocol must also be provided in the manuscript.

ChIP-seq

Data deposition

☒ Confirm that both raw and final processed data have been deposited in a public database such as GEO.

☒ Confirm that you have deposited or provided access to graph files (e.g. BED files) for the called peaks.

Data access links
May remain private before publication.

GSE171074 and GSE216989

Files in database submission

Both raw (*.fastq.gz) and processed (*.bed and *.bigwig) files are made available for download.

GSM5218278 Gar15-13 Vehicle 1 ER
GSM5218279 Gar15-13 Vehicle 2 ER
GSM5218280 Gar15-13 Vehicle 3 ER
GSM5218281 Gar15-13 Vehicle 4 ER
GSM5218282 Gar15-13 Decitabine 1 ER
GSM5218283 Gar15-13 Decitabine 2 ER
GSM5218284 Gar15-13 Decitabine 3 ER
GSM5218285 Gar15-13 Decitabine 4 ER
GSM5218286 Gar15-13 Input ER

CUT&RUN:
GSM7648680 Gar15-13 Vehicle 1 CTCF
GSM7648681 Gar15-13 Vehicle 2 CTCF
GSM7648682 Gar15-13 Vehicle 3 CTCF
GSM7648683 Gar15-13 Decitabine 1 CTCF
GSM7648684 Gar15-13 Decitabine 2 CTCF
GSM7648685 Gar15-13 Decitabine 3 CTCF
GSM7648686 Gar15-13 Vehicle 1 H3K27ac
GSM7648687 Gar15-13 Vehicle 2 H3K27ac
GSM7648688 Gar15-13 Vehicle 3 H3K27ac
GSM7648689 Gar15-13 Decitabine 1 H3K27ac
GSM7648690 Gar15-13 Decitabine 2 H3K27ac
GSM7648691 Gar15-13 Decitabine 3 H3K27ac
GSM7648692 Gar15-13 IgG

Genome browser session
(e.g. UCSC)

Hi-C and PCHi-C browser files are provided in the GEO submission. These files can be imported directly into JuiceBox and WashU Browser. ChIP-seq and CUT&RUN data generated in this paper is provided in the GEO submission.

Methodology

Replicates

Decitabine treatment was performed in two independent patient-derived xenografts (PDXs) and 7 to 8 individual PDX mice were used in therapeutic studies to obtain sufficient sample size.

EPIC DNA methylation, ER ChIP-seq and RNA-seq experiments were performed in quadruplicates and CUT&RUN, Hi-C and Promoter Capture Hi-C were performed in triplicates to assess statistical significance. Public ER ChIP-seq datasets used in this study were performed on multiple primary patient breast tumour samples as described in the respective papers.

Sequencing depth

Sequencing depth and summary statistics for all generated sequencing datasets (ChIP-seq, RNA-seq, Hi-C and PCHi-C) are provided in Supplementary Tables 2-5. For ChIP-seq datasets, each sample was sequenced in order to target a read depth of ~20+ million 75bp single-end reads. For CUT&RUN datasets, each sample was sequenced in order to target a read depth of 10 million 150bp paired-end reads.

Antibodies	Antibodies used were: Anti-Estrogen Receptor alpha (HC-20), Abcam (Cat# ab23738; RRID: AB_2104842), CTCF (CTCF CUTANA™ CUT&RUN Antibody (cat. #13-2014)), H3K27ac (Histone H3K27ac Antibody, SNAP-ChIP Certified (cat. #13-0045))
Peak calling parameters	Peaks were called with MACS2 (v2.2.6) (Zhang et al., 2008) under the default parameters (band width = 300, model fold = [5, 50], q value cutoff = 5.00e-02)
Data quality	All experiments were performed in multiple replicates. Specifically, ChIP-seq experiments were performed in four replicates for Vehicle and Decitabine-treated tumours in 2 PDX models and CUT&RUN in three replicates. All peaks are below the Macs2 FDR cut off.
Software	ChIP-seq and CUT&RUN reads were aligned against human genome (hg38/GRCh38) using bowtie2 with default parameters (--dovtail for CUT&RUN). Non-uniquely mapped, low quality (MAPQ<15) and PCR duplicate reads were removed. Peak calling of individual ChIP–seq and CT&RUN experiments was performed with MACS2 with default parameters. Statistics for each library can be found in Supplementary Table 4. Consensus peaks were identified by intersecting MACS2 peaks obtained from each sample using bedtools intersect (v.2.25.0) with min. overlap > 0.6. Differential binding analyses were performed using DiffBind (v.3.0.9) and DESeq2 (v.1.3.0) with FDR < 5%. Enrichment analyses were performed using GAT, ChIPseeker (v.1.26.0) and normalised to library size. Merged bigwig tracks for visualisation were created from merged bam files from all replicates using the bamCoverage function with scaling factor normalisation and heatmaps and average profiles were plotted with deepTools2. All code used to process and analyze ChIP-seq data is publicly available within the GitHub repository https://github.com/JoannaAch/PDX_Decitabine_3DEpigenome.
