# Supplementary material for: The potential of epigenetic therapy to target the 3D epigenome in endocrine-resistant breast cancer
Source: Nat Struct Mol Biol. 2024 Jan 5;31(3):498–512. doi: 10.1038/s41594-023-01181-7 (PMC10948365; doi:10.1038/s41594-023-01181-7)

Uncropped versions of Western blot, labelled with the relevant panel and identifying information such as the antibody used.

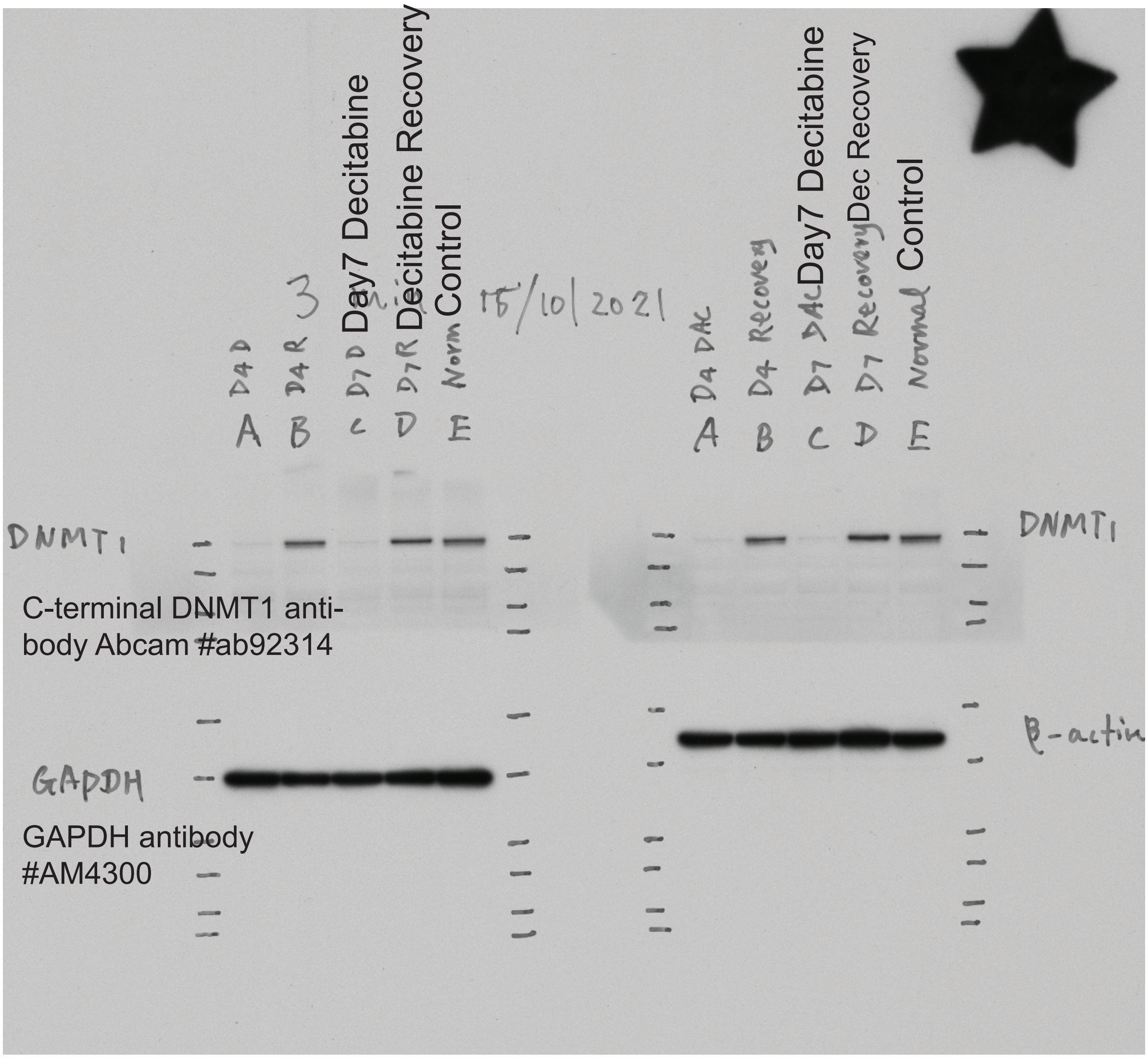

Supplement: Supplementary file 6 — Unprocessed western blots. [file 41594_2023_1181_MOESM6_ESM.pdf]
